# An Autonomous Satellite Time Synchronization System Using Remotely Disciplined VC-OCXOs

**DOI:** 10.3390/s150817895

**Published:** 2015-07-23

**Authors:** Xiaobo Gu, Qing Chang, Eamonn P. Glennon, Baoda Xu, Andrew G. Dempseter, Dun Wang, Jiapeng Wu

**Affiliations:** 1 School of Electronic and Information Engineering, Beihang University, Xueyuan Road No. 37, Haidian District, Beijing 100191, China; E-Mails: changq@263.net (Q.C.); xubaoda@foxmail.com (B.X.); 2 Australian Centre for Space Engineering Research (ACSER), School of Electrical Engineering and Telecommunications, University of New South Wales, High Street, Sydney, NSW 2052, Australia; E-Mails: e.glennon@unsw.edu.au (E.P.G.); a.dempster@unsw.edu.au (A.G.D.); 3 Space Star Technology Co., Ltd., Zhichun Road No. 82, Haidian District, Beijing 100191, China; E-Mails: dawun04@163.com (D.W.); wjp82-12-13@163.com (J.W.)

**Keywords:** time synchronization, dual one-way ranging, inter-satellite link, clock adjustment

## Abstract

An autonomous remote clock control system is proposed to provide time synchronization and frequency syntonization for satellite to satellite or ground to satellite time transfer, with the system comprising on-board voltage controlled oven controlled crystal oscillators (VC-OCXOs) that are disciplined to a remote master atomic clock or oscillator. The synchronization loop aims to provide autonomous operation over extended periods, be widely applicable to a variety of scenarios and robust. A new architecture comprising the use of frequency division duplex (FDD), synchronous time division (STDD) duplex and code division multiple access (CDMA) with a centralized topology is employed. This new design utilizes dual one-way ranging methods to precisely measure the clock error, adopts least square (LS) methods to predict the clock error and employs a third-order phase lock loop (PLL) to generate the voltage control signal. A general functional model for this system is proposed and the error sources and delays that affect the time synchronization are discussed. Related algorithms for estimating and correcting these errors are also proposed. The performance of the proposed system is simulated and guidance for selecting the clock is provided.

## 1. Introduction

Some existing satellite time synchronization systems, such as the gravity recovery and climate experiment (GRACE) achieve synchronization by means of compensating the clock errors at the ground station rather than producing the synchronized and syntonized timing signals [1,2]. Achieving real-time synchronization and syntonization of satellite systems should produce more ideal signals and eliminate the various errors due to the inaccuracy of the on-board clocks.

In general, atomic clocks have better long-term stability but also worse short-term stability, greater volume, weight, power, price and shorter lifetime compared with a high quality crystal oscillator (although chip-scale atomic clocks have been developed, their stability is not as good as full-scale atomic clocks and they are not space qualified). For this reason, global navigation satellite systems (GNSS), such as the global positioning system (GPS), adopts a time-keeping system (TKS) that couples the atomic clock with a voltage-controlled crystal oscillator (VCXO) on-board the satellite to produce the reference timing signal [3,4]. This sophisticated and expensive military-based system is not suitable when low cost and low complexity is required. The Japanese Quasi-Zenith Satellite System proposed a solution that they refer to as “remote time synchronization system for the on-board crystal oscillator” (RESSOX) to realize time synchronization between the ground station and satellites [5,6]. Different from GPS, the QZS employs a VCXO as the on-board clock in the RESSOX scheme. This VCXO is remotely steered by an atomic clock that is located in the ground station by means of a series of feed-forward control and feedback control. The positioning accuracy of RESSOX is better than using GPS & QZSS with on-board atomic clock [7]. However, it was not implemented in the final QZSS owing to the budget constraints. 

The systems mentioned above are steered by ground stations, which means the redundant ground stations and personnel are essential. Autonomous time synchronization for space systems is put forward in recent years. In [8], the NAMURU V3.2 spaceborne receiver, which is developed specifically for CubeSat formation flying, is disciplined to an external reference-GPS time. However, this kind of one-way time dissemination method has limited precision (20 nanoseconds). Hence starting from Block IIR, GPS adopts inter-satellite links combined with the polling time division duplex scheme (PTDD) to achieve autonomous time synchronization in cases where the ground station is not available [9,10]. Similar discussions about other GNSS systems could be found in [11,12], although these are not actually being implemented yet. In addition, some other scientific-based space missions are also being deployed, In [13], a method based on asynchronous two way time-stamping exchange is being employed, where the clock skews and clock offsets are estimated but not adjusted. 

Based on the above discussions, when spacecraft activities require autonomous, robust, high-accuracy time synchronization performance, the question regarding how to reduce overall satellite cost, power consumption, on-board weight and volume, and improve satellite lifespan becomes an interesting issue. In this paper, we propose a remote physical time synchronization system based on a VC-OCXO. The main contributions of this paper are threefold. First, it has the STDD & FDD configuration, in which the satellites simultaneously communicate with each other via different frequencies. Different from the PTDD adopted by GPS crosslink, STDD has the advantages on ranging precision, clock synchronization precision, ranging efficiency and channel utilization ratio [14]. Second, a functional model for the synchronization loop that contains clock error measurement, prediction and adjustment, is designed to be applicable to various space scenarios. A relative motion compensation method based on the proposed model is provided for this purpose. Unlike the LS based relative motion compensation methods that makes use of pseudo-range, carrier phase and even Doppler observations [15,16], the proposed method only requires pseudo-range observations and ephemeris information, and the error originates from relative motion could be calculated by means of one communication link. Third, it proposes a cost-effective and energy-effective way to achieve the desired synchronization and syntonization performance of satellite systems.

The paper is organized as follows: in Section 2 we propose an autonomous time-synchronization system and illustrate the design in detail. In Section 3, a software-defined simulator is presented to investigate the performance of this system. Related simulation results are provided and analyzed. Finally, Section 4 concludes the paper and describes the advantages and disadvantages compared with existing time synchronization systems. Recommendations for further improvement are also provided.

## 2. System Design

The proposed system is a Master/Slave architecture, with the aim of the system to synchronize the VC-OCXO with a remote reference clock. GNSS-like technology is adopted as a basic element to establish the communication network. The master clock is considered as the reference clock and is assumed absolutely accurate and stable. It could be situated either on another satellite or at the ground station. For the sake of simplification, we call it the master satellite in this paper. 

**Figure 1:**
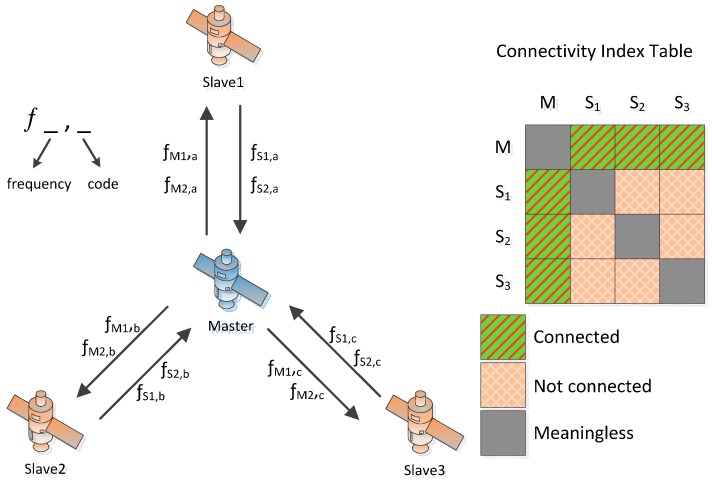
An example of connectivity with three slave satellites and a master satellite.

The slave satellite carries an on-board VC-OCXO as its frequency source. More than one slave satellite may be employed in this system, which means it has a centralized topology. As the STDD & FDD configuration is employed, each slave satellite and the master satellite simultaneously intercommunicates with each other in defined time slots in terms of the CDMA & frequency division multiple access (FDMA) channel, which is different from the PTDD and broadcasting scheme adopted in GPS crosslink. An example of this system is illustrated in Figure 1, which also includes a table showing the connectivity between each of the elements. There are two fundamental scenarios for this system: ground to satellite mode (GSM) and satellite to satellite mode (SSM). Each of the two scenarios needs to be discussed separately due to the differences in parameters such as baseline length, velocity, communication frequency, visibility and space environment. 

**Figure 2:**
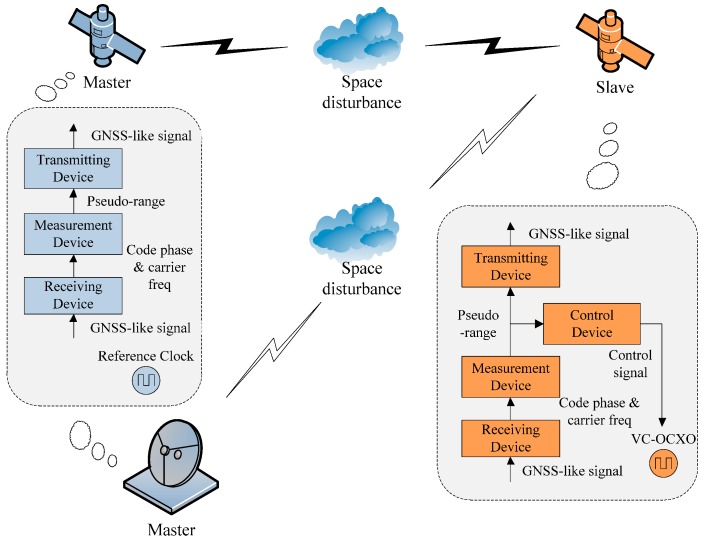
Simplified representation of this autonomous time synchronization system. Note that only one master is present, although this may be space or ground based.

Figure 2 shows the simplified representation of this system. The receiving device receives the GNSS like signal and after down conversion, the intermediate frequency (IF) is quantized using an analogue to digital converter (ADC). In the Measurement Device, signal acquisition, tracking and decoding processes are included for data recovery, code and carrier phase extraction, while measurement of pseudoranges and pseudorange-rates is also performed. The phase shift is measured by the comparison of the pseudo-noise (PN) code of the master satellite and the slave satellite. The transmitting device transmits the GNSS-like signal that incorporates clock error information by a series of processes that include a digital signal generator, digital to analogue converter (DAC), up-converter and amplifier. Different from the master satellite, the slave satellite carries not only a transmitting device, a receiving device and a measurement device, but also a control device. It is used to process the range measurement information from two satellites. The phase shift that is caused by the clock error is extracted by means of delay correction, after which the voltage control signal is generated to adjust the voltage of VC-OCXO.

In addition, it is noted that this physical time synchronization method could be considered as a closed loop. The clock error of the slave satellite relative to the master satellite is extracted from pseudo-range measurement. It is translated into the control signal, which is used to adjust the VC-OCXO of the slave satellite. Meanwhile, the corrected VC-OCXO generates the timing signal for ranging measurement activities. Therefore, the scheme could be divided into two parts: clock error extraction and clock adjustment, which are the two fundamental issues we consider. 

**Figure 3:**
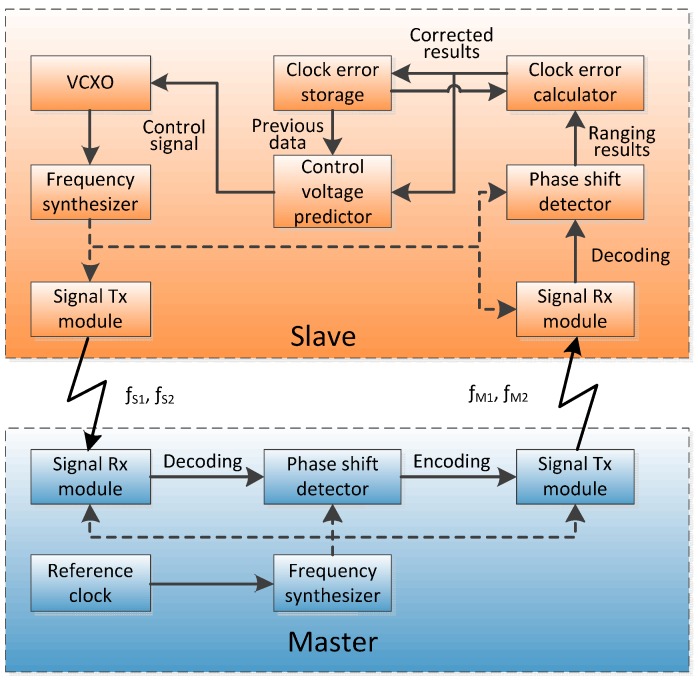
Schematic of the proposed system.

In order to solve these two problems, a specific software simulator based on dual one-way ranging measurements and PLL [17] has been developed. The following is the principle of this new method, as shown in Figure 3. 

Based on the discussions above, the differences between the proposed system and the existing similar systems can be presented, as shown in Table 1.

**Table 1:** Comparison of synchronization systems.

System	Clock	Clock Error Extraction Method	Syntonization	Autonomous
GPS Block IIR crosslink	Atomic clock & VCXO	Asynchronous two-way ranging	Yes	Yes
RESSOX	VC-OCXO	Using GPS signals and the follow-up processes on the ground station	Yes	No
NAMURU V3.2	VC-TCXO	Disciplining to GPS time	Yes	Yes
GRACE	OCXO	Synchronous dual one-way ranging	No	No
Proposed system	VC-OCXO	STDD & FDD dual one-way ranging	Yes	Yes

### 2.1. Clock Model

Owing to the reference clock being considered as a perfect frequency source, we only need to model the VC-OCXO on-board the slave satellite. The instantaneous voltage of the VC-OCXO can be modeled as:

(1) $$V_{0}(t)=(A_{0}+\text{ε}(t))\sin(2\text{π}f_{0}t+\phi (t))$$

where *t* is the reference time generated by the master clock, *A*_0_ and *f*_0_ are the nominal amplitude and frequency values of the signal respectively, and ε(t) and ϕ(t) correspond to the amplitude and phase random fluctuations [18,19]. 

The instantaneous frequency is:

(2) $$f\left(t\right)=\frac{1}{2\text{π}}\frac{d}{dt}\left(2\text{π}f_{0}t+\phi \left(t\right)\right)=f_{0}+\frac{1}{2\text{π}}\frac{d}{dt}\phi \left(t\right)$$

The phase and frequency deviations are expressed as:

(3) $$x\left(t\right)=\frac{\phi \left(t\right)}{2\text{π}f_{0}}$$

(4) $$y\left(t\right)=\frac{f\left(t\right)-f_{0}}{f_{0}}=\frac{1}{2\text{π}f_{0}}\frac{d}{dt}\phi \left(t\right)$$

The quantity x(t) is the clock error between two clocks. It is usually modeled by Equation (5) [20]:

(5) $$x\left(t\right)=a_{0}+a_{1}t+\frac{1}{2}a_{2}t^{2}+\text{ψ}\left(t\right)$$

where $a_{0}$, $a_{1}$ and $a_{2}$ represent the clock bias, clock drift and clock drift rate respectively, while ψ(t) denotes the random noise, which can be expressed by the sum of five independent noise terms [21]. Its power-law spectral density is represented as:

(6) $$S_{y}(f)=h_{-2}f^{-2}+h_{-1}f^{-1}+h_{0}f^{0}+h_{1}f^{1}+h_{2}f^{2}=\sum_{\text{α}=-2}^{2}h_{\text{α}}f^{\text{α}}$$

These five random noise terms are known in the metrological literature as:

α=−2 : random walk frequency modulation (RWFM).

α=−1 : flicker frequency modulation (FFM).

α=0 : white frequency modulation (WFM).

α=1 : flicker phase modulation (FPM).

α=2 : white phase modulation (WPM).

Oscillator performance also can be described in time domain by using the Allan variance:

(7) $${\text{σ}}_{y}^{2}(n{\text{τ}}_{0})=\langle {\left({\overset{-}{y}}_{i+1}-{\overset{-}{y}}_{i}\right)}^{2}\rangle =\frac{1}{2\left(M-1\right)}\overset{M-1}{\underset{i=1}{\sum }}{\left({\overset{-}{y}}_{i+1}-{\overset{-}{y}}_{i}\right)}^{2}$$

Here the operator 〈·〉 represents time averaging, and *M* is the observation interval, *y_i_* is the *i* th of *M* fractional frequency values averaged over the measurement (sampling) interval [22]. We adopt the wavelet transform algorithm to realize the simulation of phase noise [23]. 

### 2.2. Ranging Measurement

**Figure 4:**
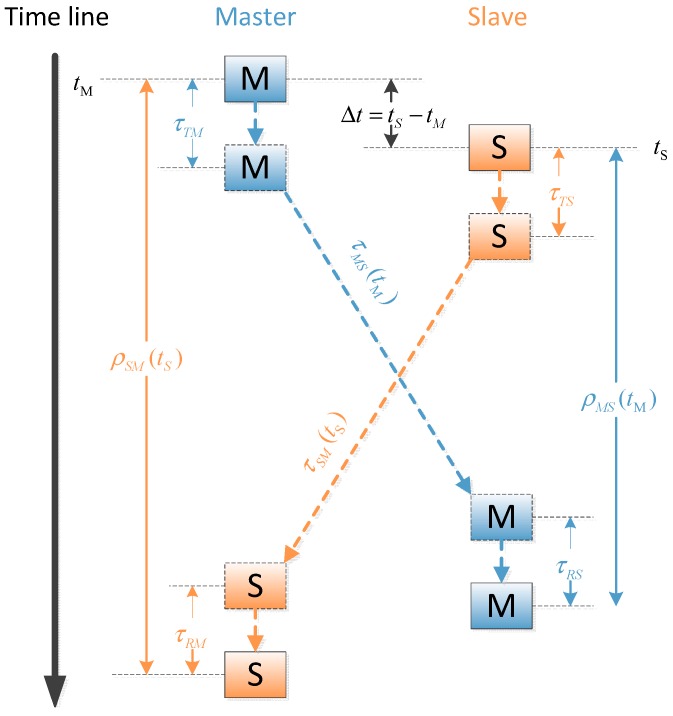
Dual one-way ranging measurements, where the boxes with M & S denote master and slave packets.

Figure 4 shows the principle of dual one way ranging measurement. Unlike the existing ranging method employed by GPS crosslink, the master satellite and the slave satellite communicate with each other simultaneously, but it is a “pseudo-simultaneous” scheme because of the clock skew and clock offset between two clocks. The parameters are defined as follows: Δt is the initial clock error between the master clock and the slave clock, ${\text{τ}}_{TM}$, ${\text{τ}}_{RM}$, ${\text{τ}}_{TS}$, ${\text{τ}}_{RS}$ are the transmitting and receiving delay of master satellite and slave satellite, respectively. ${\text{τ}}_{MS}(t_{M})$ and ${\text{τ}}_{SM}(t_{S})$ are the signal propagation delays. 

Hence the pseudo-ranges measured from each side and illustrated in Figure 4 are represented as:

(8) $${\text{ρ}}_{SM}(t_{S})=\text{Δ}t\left(t_{M}\right)+{\text{τ}}_{SM}\left(t_{S}\right)+{\text{τ}}_{TS}+{\text{τ}}_{RM}\text{+}I_{SM}+d_{SM}$$

(9) $${\text{ρ}}_{MS}(t_{M})=-\text{Δ}t\left(t_{M}\right)+{\text{τ}}_{MS}\left(t_{M}\right)+{\text{τ}}_{TM}+{\text{τ}}_{RS}+{\text{ε}}_{\tau S}+I_{MS}+d_{MS}$$

where *I* denotes the ionospheric delay between *S* and *M*, *d* denotes the other delay effects including tropospheric delay and Sagnac delay, *c* is the speed of light, ${\text{ε}}_{\tau S}$ is the clock bias of the slave clock, which is derived from the ranging measurement process. From Equation (5), ${\text{ε}}_{\tau S}$ can be expressed as:

(10) $$\begin{array}{l} {\text{ε}}_{\tau S}=\int_{{\text{τ}}_{MS\left(t_{M}\right)}+{\text{τ}}_{TM}+{\text{τ}}_{RS}}\left(\frac{v_{S}\left(t\right)-v_{0}}{v_{0}}\right)dt\\ =y\left(t_{M}\right)\left({\text{τ}}_{MS\left(t_{M}\right)}+{\text{τ}}_{TM}+{\text{τ}}_{RS}\right)+\frac{1}{2}a_{2}{\left(t_{M}+{\text{τ}}_{MS\left(t_{M}\right)}+{\text{τ}}_{TM}+{\text{τ}}_{RS}\right)}^{2} \end{array}$$

where $y(t_{M})$ is the frequency deviations at the instance $t_{M}$. Unlike the $a_{1}$ presented in Equation (5), it is not expressed in terms of constants because the feedback control varies the characteristic of the slave clock. Without loss of generality, assuming a total transmission time of 200 milliseconds and $y(t_{M})$ and $a_{2}$ are 1 × 10^−12^/s and 1 × 10^−18^/s^2^ separately, then ${\text{ε}}_{\tau S}$ is approximately 20 picoseconds, which can be ignored.

Differencing and summing Equations (8) and (9) yields the instantaneous clock error and the instantaneous relative distance between two satellites:

(11) $$\text{Δ}t\left(t_{M}\right)=\frac{{\text{ρ}}_{SM}(t_{S})-{\text{ρ}}_{MS}(t_{M})}{2c}-\frac{{\text{τ}}_{SM}\left(t_{S}\right)-{\text{τ}}_{MS}\left(t_{M}\right)}{2}-\frac{{\text{τ}}_{TM}+{\text{τ}}_{RS}-{\text{τ}}_{TS}-{\text{τ}}_{RM}}{2}-\frac{I_{SM}-I_{MS}}{2}-\frac{{\text{ε}}_{\tau S}}{2}-\frac{d_{SM}-d_{MS}}{2}$$

(12) $$\begin{array}{ll} D\left(t_{M}\right) & \approx \frac{{\text{ρ}}_{SM}(t_{S}){\text{+ρ}}_{MS}(t_{M})}{2}\\ & =\left(\frac{{\text{τ}}_{SM}\left(t_{S}\right){\text{+τ}}_{MS}\left(t_{M}\right)}{2}+\frac{{\text{τ}}_{TM}+{\text{τ}}_{RS}{\text{+τ}}_{TS}{\text{+τ}}_{RM}}{2}+\frac{I_{SM}+I_{MS}}{2}+\frac{d_{SM}+d_{MS}}{2}+\frac{{\text{ε}}_{\tau S}}{2}\right)\cdot c \end{array}$$

The first term of Equation (11) can be obtained directly from the ranging measurement of each satellite. The second term denotes the relative motion error. It is easy to understand that Equation (12) makes sense when the master satellite and the slave satellite are stationary, but $D\left(t_{M}\right)$ would not equal to the true baseline if there is a relative motion between the two satellites, owing to the changes of ${\text{τ}}_{MS}(t_{M})$ and ${\text{τ}}_{SM}(t_{S})$. This is the reason why Equation (12) uses an approximately equal. It implies that the error would be critical if the system members have a fast relative velocity between each other. Take GNSS for example, the maximum time synchronization error that originates from relative motion can be shown to be approximately 1 microsecond. The related equation will be presented later in this section. The third term can be calibrated precisely before the electronic apparatus employed in the synchronization loop are installed into the payload, but is expected to be within 0.1 nanosecond. The fourth term can be effectively compensated through the use of the dual frequency ionospheric correction algorithm, which is described in [24] as:

(13) $$R=\frac{f_{1}^{2}R_{1}-{f_{2}}^{2}R_{2}}{f_{1}^{2}-f_{2}^{2}}$$

where *f_i_* is the radio frequency, and *R_i_* is the corresponding pseudo-range measurement. Certain frequencies can be adopted for different scenarios and goals of synchronization precision. The fifth term can be obtained by means of Equation (10), although the on-board clocks normally have good stabilities and as such, this term only has a small effect during a short time interval. The sixth term, which denotes the other delays, include the tropospheric delay and Sagnac delay in the GSM. The European Geostationary Navigation Overlay Service (EGNOS) tropospheric correction model can be employed for the GSM to eliminate most of the tropospheric delay [25]. Meanwhile, a Sagnac-effect correction method can be applied to the propagation time of the signal in such a case [26]. 

**Figure 5:**
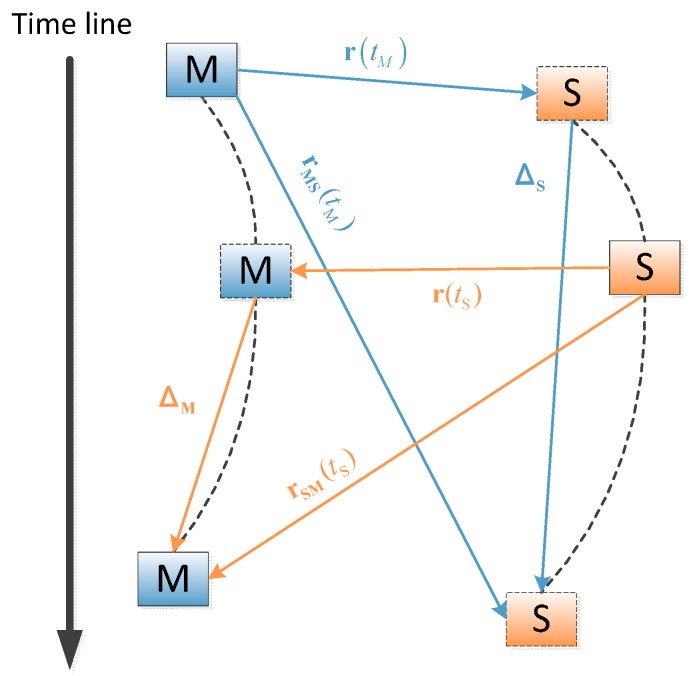
Changes of the position of the two satellites during the signal propagation period.

An applicable solution for this specific requirement is proposed in this paper. The relative motion error consists of two parts, namely the ephemeris error and the Doppler shift. In the proposed system, we assume that the slave satellites only have the pseudo-range observation and the ephemeris of the entire system members. Figure 5 shows how the positions of the master satellite and the slave satellite change. We define $r\left(t_{i}\right)$ as the displacement from the satellite i to the other satellite at $t_{i}$, while $r_{SM}\left(t_{i}\right)$ and $r_{MS}\left(t_{i}\right)$ are the displacements from satellite M/S to satellite S/M during the period of $\tau_{SM}\left(t_{i}\right)$ and $\tau_{MS}\left(t_{i}\right)$, such as $r_{SM}\left(t_{i}\right)=\tau_{SM}\left(t_{i}\right)\cdot c$, $r_{MS}\left(t_{i}\right)=\tau_{MS}\left(t_{i}\right)\cdot c$. They are modeled by:

(14) $$\left|r\left(t_{S}\right)\right|=f\left(r_{SM},\Delta_{M}\right)=\sqrt{{\left(r_{SM}-\Delta_{M}\right)}^{T}\left(r_{SM}-\Delta_{M}\right)}$$

where Δ_**M**_ denotes the change of position from the instance that the slave satellite transmits the ranging signal to the instance that the master satellite receives the signal. It can be expressed as Δ_**M**_ = $v_{M}\cdot \tau_{SM}\left(t_{s}\right)$, where **v_M_** is the velocity of the master satellite, which varies over time. 

Equation (14) can be expanded by the first-order Taylor series as:

(15) $$\begin{array}{cc} \left|r(t_{S})\right| & \approx {f|}_{{\text{Δ}}_{M}=0}+{\frac{\partial f}{\partial {\text{Δ}}_{M}}|}_{{\text{Δ}}_{M}=0}\cdot {\text{Δ}}_{M}\\ & =\sqrt{r_{SM}{\left(t_{S}\right)}^{T}r_{SM}(t_{S})}+{\frac{\left({\text{Δ}}_{M}-r_{SM}(t_{S})\right)}{\sqrt{{\left[r_{SM}(t_{S})-{\text{Δ}}_{M}\right]}^{T}\left[r_{SM}(t_{S})-{\text{Δ}}_{M}\right]}}|}_{{\text{Δ}}_{M}=0}\cdot {\text{Δ}}_{M}\\ & =\left|r_{SM}(t_{S})\right|-{e_{SM}}^{T}{\text{Δ}}_{M}\\ & =\left|r_{SM}(t_{S})\right|-{e_{SM}}^{T}\cdot \int_{{\text{τ}}_{SM}(t_{S})}v_{LOS}^{M}(t)dt\\ & \approx \left|r_{SM}(t_{S})\right|-{\text{τ}}_{SM}(t_{S})\cdot {e_{SM}}^{T}\cdot {\bar{v}}_{LOS}^{M} \end{array}$$

**Figure 6:**
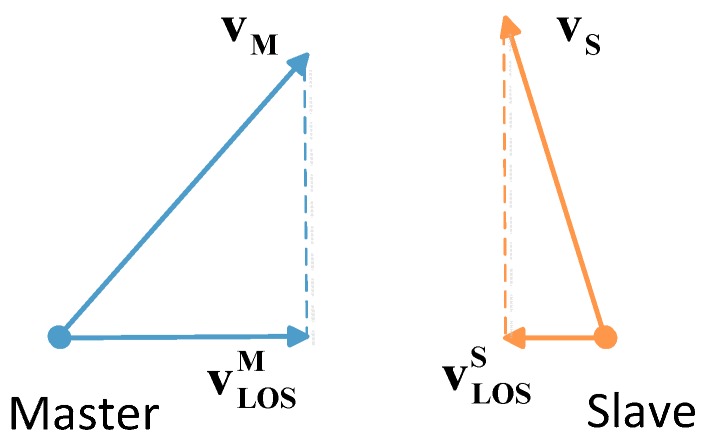
Diagrammatic sketch of LOS velocity.

Similarly:

(16) $$\begin{array}{cc} \left|r(t_{M})\right| & \approx {f|}_{{\text{Δ}}_{S}=0}+{\frac{\partial f}{\partial {\text{Δ}}_{S}}|}_{{\text{Δ}}_{S}=0}\cdot {\text{Δ}}_{S}\\ & =\sqrt{r_{MS}{\left(t_{M}\right)}^{T}r_{MS}(t_{M})}+{\frac{\left({\text{Δ}}_{S}-r_{MS}(t_{M})\right)}{\sqrt{{\left[r_{MS}(t_{M})-{\text{Δ}}_{S}\right]}^{T}\left[r_{MS}(t_{M})-{\text{Δ}}_{S}\right]}}|}_{{\text{Δ}}_{S}=0}\cdot {\text{Δ}}_{S}\\ & =\left|r_{MS}(t_{M})\right|-{e_{MS}}^{T}{\text{Δ}}_{S}\\ & =\left|r_{MS}(t_{M})\right|-{e_{MS}}^{T}\cdot \int_{{\text{τ}}_{MS}(t_{M})}v_{LOS}^{S}(t)dt\\ & \approx \left|r_{MS}(t_{M})\right|-{\text{τ}}_{MS}(t_{M})\cdot {e_{MS}}^{T}\cdot {\bar{v}}_{LOS}^{S} \end{array}$$

where $v_{LOS}^{i}$ denotes the line of sight (LOS) velocity of satellite *i* (Figure 6), ${\bar{v}}_{LOS}^{i}$ denotes the average of $v_{LOS}^{i}$ during the period of signal propagation, **e_ij_** denotes the unit vector of **r_ij_** (*t_i_*), **e_ij_** = −**e_ij_**. 

Furthermore, the error caused by clock error can be expressed as:

(17) $$\begin{array}{cc} \text{Δτ} & =\text{τ}(t_{M})-\text{τ}(t_{S})\\ & =\frac{1}{c}\cdot \int_{\text{Δ}t}[v_{LOS}^{M}{\left(t\right)}^{T}\cdot e_{SM}+v_{LOS}^{S}{\left(t\right)}^{T}\cdot e_{MS}]dt \end{array}$$

where *τ*(*t_M_*) = |**r**(*t_M_*)|/*c*, *τ*(*t_s_*) = |**r**(*t_s_*)|/*c*. Substitution of Equations (15)–(17) into Equations (11) and (12) yields:

(18) $$\left\{ \begin{array}{c} \text{τ}(t_{S})=\frac{1}{2}\cdot \left[\frac{{\text{ρ}}_{SM}(t_{S})}{c}+\frac{{\text{ρ}}_{MS}(t_{M})}{c}-\text{Δτ}\cdot \frac{c}{c-{e_{SM}}^{T}\cdot {\bar{v}}_{LOS}^{S}}-{\text{Ω}}_{+}\right]\cdot c^{\prime }\\ \text{Δ}t=\frac{1}{2}\cdot \left[\frac{{\text{ρ}}_{SM}(t_{S})}{c}-\frac{{\text{ρ}}_{MS}(t_{M})}{c}+\text{τ}(t_{S})\cdot c^{\prime \prime }+\text{Δτ}\cdot \frac{c}{c-{e_{SM}}^{T}\cdot {\bar{v}}_{LOS}^{S}}-{\text{Ω}}_{-}\right] \end{array}\right.$$

where:

(19) $$\{\begin{array}{c} {\text{Ω}}_{+}={\text{τ}}_{TS}+{\text{τ}}_{RM}\text{+}I_{S}+d_{S}+{\text{τ}}_{TM}+{\text{τ}}_{RS}+{\text{ε}}_{\tau M}+I_{M}+d_{M}\\ {\text{Ω}}_{-}={\text{τ}}_{TS}+{\text{τ}}_{RM}\text{+}I_{S}+d_{S}-{\text{τ}}_{TM}-{\text{τ}}_{RS}-{\text{ε}}_{\tau M}-I_{M}-d_{M}\\ c^{\prime }=2\cdot {\left[\frac{c}{c-{e_{MS}}^{T}\cdot {\bar{v}}_{LOS}^{S}}+\frac{c}{c-{e_{SM}}^{T}\cdot {\bar{v}}_{LOS}^{M}}\right]}^{-1}\\ c^{\prime \prime }=\frac{c}{c-{e_{MS}}^{T}\cdot {\bar{v}}_{LOS}^{S}}-\frac{c}{c-{e_{SM}}^{T}\cdot {\bar{v}}_{LOS}^{M}} \end{array}$$

The LOS velocity of the satellites changes slowly in a short time interval, thus we can assume:

(20) $$\{\begin{array}{c} {\bar{v}}_{LOS}^{M}=v_{LOS}^{M}(t_{M})=v_{LOS}^{M}(t_{S})\\ {\bar{v}}_{LOS}^{S}=v_{LOS}^{S}(t_{M})=v_{LOS}^{S}(t_{S}) \end{array}$$

Therefore, the terms $c^{\prime }$ and $c^{\prime \prime }$ can be given by:

(21) $$\begin{array}{cc} c^{\prime } & =1-\frac{1}{2}\cdot \frac{{e_{MS}}^{T}\cdot {\bar{v}}_{LOS}^{S}+{e_{SM}}^{T}\cdot {\bar{v}}_{LOS}^{M}}{c}-\frac{\frac{{\left({e_{MS}}^{T}\cdot {\bar{v}}_{LOS}^{S}-{e_{SM}}^{T}\cdot {\bar{v}}_{LOS}^{M}\right)}^{2}}{4c^{2}}}{1+\frac{1}{2}\cdot \frac{{e_{MS}}^{T}\cdot {\bar{v}}_{LOS}^{S}+{e_{SM}}^{T}\cdot {\bar{v}}_{LOS}^{M}}{c}}\\ & \approx 1-\frac{1}{2}\cdot \frac{{e_{MS}}^{T}\cdot {\bar{v}}_{LOS}^{S}+{e_{SM}}^{T}\cdot {\bar{v}}_{LOS}^{M}}{c}\\ & =1-\frac{1}{2}\cdot \frac{{e_{MS}}^{T}\cdot \left({\bar{v}}_{LOS}^{S}-{\bar{v}}_{LOS}^{M}\right)}{c} \end{array}$$

(22) $$\begin{array}{ll} c^{\prime \prime } & =\frac{c}{c-{e_{MS}}^{T}\cdot {\bar{v}}_{LOS}^{S}}-\frac{c}{c-{e_{SM}}^{T}\cdot {\bar{v}}_{LOS}^{M}}\\ & =\frac{c\left({e_{MS}}^{T}\cdot {\bar{v}}_{LOS}^{S}-{e_{SM}}^{T}\cdot {\bar{v}}_{LOS}^{M}\right)}{\left(c-{e_{MS}}^{T}\cdot {\bar{v}}_{LOS}^{S}\right)\left(c-{e_{SM}}^{T}\cdot {\bar{v}}_{LOS}^{M}\right)}\\ & \text{≈}\frac{{e_{MS}}^{T}\cdot {\bar{v}}_{LOS}^{S}-{e_{SM}}^{T}\cdot {\bar{v}}_{LOS}^{M}}{c}\\ & =\frac{{e_{MS}}^{T}\cdot \left({\bar{v}}_{LOS}^{S}+{\bar{v}}_{LOS}^{M}\right)}{c} \end{array}$$

Substitution of Equations (21) and (22) into Equation (18) yields:

(23) $$\{\begin{array}{c} \text{τ}(t_{S})=\frac{1}{2}\cdot \left[\frac{{\text{ρ}}_{SM}(t_{S})}{c}+\frac{{\text{ρ}}_{MS}(t_{M})}{c}-\text{Δτ}\cdot \frac{c}{c-{e_{MS}}^{T}\cdot {\bar{v}}_{LOS}^{S}}-{\text{Ω}}_{-}\right]\cdot \left[1-\frac{1}{2}\cdot \frac{{e_{MS}}^{T}\cdot \left({\bar{v}}_{LOS}^{S}-{\bar{v}}_{LOS}^{M}\right)}{c}\right]\\ \text{Δ}t=\frac{1}{2}\cdot \left[\frac{{\text{ρ}}_{SM}(t_{S})}{c}-\frac{{\text{ρ}}_{MS}(t_{M})}{c}+\text{τ}(t_{S})\cdot \frac{{e_{MS}}^{T}\cdot \left({\bar{v}}_{LOS}^{S}+{\bar{v}}_{LOS}^{M}\right)}{c}+\text{Δτ}\cdot \frac{c}{c-{e_{MS}}^{T}\cdot {\bar{v}}_{LOS}^{S}}-{\text{Ω}}_{+}\right] \end{array}$$

Therefore, the maximum ranging error and clock error of slave satellite owing to relative motion can be expressed as:

(24) $$\{\begin{array}{c} \text{Δ}R_{RM\max}=-\text{τ}(t_{S})\cdot \frac{{\left\{{e_{MS}}^{T}\cdot \left({\bar{v}}_{LOS}^{S}-{\bar{v}}_{LOS}^{M}\right)\right\}}_{\max}}{2}\\ \text{Δ}t_{RM\max}=\text{τ}(t_{S})\cdot \frac{{\left\{{e_{MS}}^{T}\cdot \left({\bar{v}}_{LOS}^{S}+{\bar{v}}_{LOS}^{M}\right)\right\}}_{\max}}{c} \end{array}$$

### 2.3. Adjustment Method

The pseudo-ranges in the first term of Equation (11) are measured on each satellite, which means the information must be collected on the slave satellite for subsequent processing. In other words, the pseudo-ranges obtained from the master satellite must be packed into the data frames and transmitted to the slave satellite. Here we propose using the Proximity-1 Version-3 Space Link Protocol as defined by the Consultative Committee for Space Data Systems (CCSDS) for data frame design [27]. With a data overlay of 1000 bits/s and 200 bits per sub frame, the minimum clock adjustment time period would be 0.2 s. The processor on the slave satellite extrapolates the previous clock errors with a nonlinear LS method. The time to be adjusted is given by a third-order PLL, which is then converted into the voltage control signal. The on-board VC-OCXOs of the slave satellites change the frequencies at the fixed time period:

(25) $$\text{Δ}t\left(t_{i}+t\right)=x\left(t_{i}\right)+\left(y\left(t_{i}\right)\text{+}f_{fb}\left(t_{i}\right)\right)t+\frac{1}{2}a_{2}{\left(t_{i}+t\right)}^{2}+\text{ψ}\left(t_{i}+t\right),0\leq t<t_{i}-t_{i-1}$$

where *t_i_* is the *i*th clock adjustment instance, *f_fb_* is the adjustment frequency converted by the voltage control signal. 

**Figure 7:**
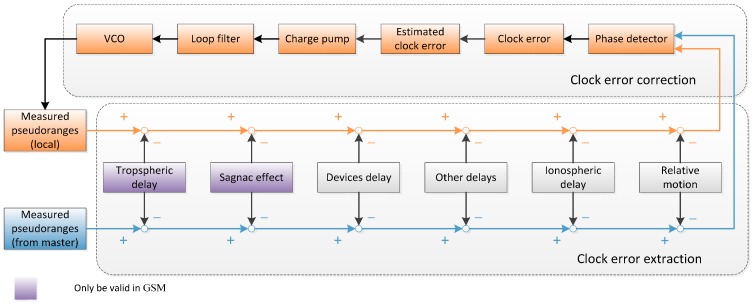
Schematic of the proposed clock adjustment algorithms on a slave satellite.

As shown in Figure 7, a series of phase delay elimination procedures are implemented before the slave clock starts detecting the phase deviation between two clocks. It is noted that different effects should be considered in different scenarios, due to the space condition, separation distance and relative velocity. Equation (24) determines whether it is necessary to compensate for the relative motion error. The delay elimination methods for each kind of delays are represented in Table 2.

**Table 2:** Different delays and corresponding correction methods.

Delays	Correction Method
**Ionospheric delay**	Dual-frequency correction
**Device delay**	Pre-calibration
**Relative motion delay**	Relative motion compensation
**Ideal propagation delay**	Dual one way ranging measurement
Delays only exist in GSM	**Correction method**
**Tropospheric delay** **Sagnac effect**	EGNOS tropospheric correction model Sagnac correction

## 3. Case Study and Simulation

A Maltab software simulation was developed to fully explore the performance of the system. We simulated four slave clocks with different stabilities. The Allan deviation of each phase noise component at *τ* = 1 s is shown in Table 3, and the design of the PLL control loop is shown in Table 4.

**Table 3:** Allan deviation of each phase noise component at *τ* = 1 s.

Number	Designation	RWFM	FFM	WFM	FPM	WPM
1	σ at τ=1s	1.2 × 10^−11^	1.0 × 10^−10^	1.0 × 10^−10^	1.0 × 10^−9^	1.0 × 10^−9^
2	σ at τ=1s	1.2 × 10^−12^	1.0 × 10^−11^	1.0 × 10^−11^	1.0 × 10^−10^	1.0 × 10^−10^
3	σ at τ=1s	1.2 × 10^−13^	1.0 × 10^−12^	1.0 × 10^−12^	1.0 × 10^−11^	1.0 × 10^−11^
4	σ at τ=1s	1.2 × 10^−14^	1.0 × 10^−13^	1.0 × 10^−13^	1.0 × 10^−12^	1.0 × 10^−12^

**Table 4:** Simulation parameters of PLL.

Order of PLL	Parameters
**Third order**	*a*_3_ = 1.1
	*b*_3_ = 2.4
	$B_{L}=(a_{3}b_{3}^{2}+a_{3}^{2}-b_{3})\cdot {\text{ω}}_{n}/4(a_{3}b_{3}-1)$

### 3.1. Relative Motion Compensation

**Figure 8:**
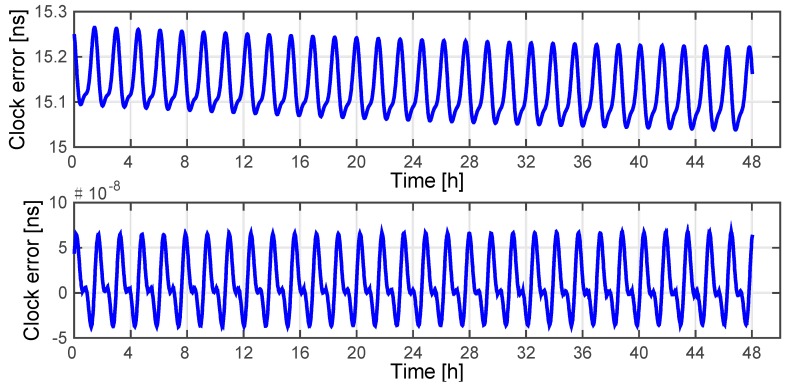
Decoupling clock error of GRACE 1 before (up) and after (down) motion compensation.

**Figure 9:**
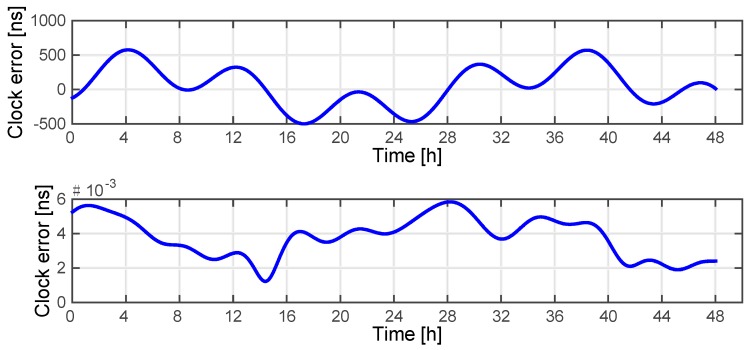
Decoupling clock error of Beidou A2 before (up) and after (down) motion compensation.

In this Matlab simulation, since the slave satellites are independent of each other, we choose a master satellite and a slave satellite to perform the simulation. The orbit data from the GRACE mission and Beidou G2 & Beidou A2 were employed via Satellite Tool Kit (STK) because they have significantly different inter-satellite baselines and relative velocities. For both scenarios, we assume that the measurement noise is zero, and the delays including the ionospheric delay, device delay and other delays are effectively eliminated by corresponding methods represented in Table 1. The reason for this is that we want to focus on the theoretical performance of the considered relative motion compensation scheme, rather than on the degrading effects of the measurement noise and the residuals. The positioning error of the receiver is assumed to contribute 1 picosecond of timing error. The maximum decoupling error caused by relative motion can be obtained from Equation (24), which if sufficiently large can be corrected using Equation (23). The compensation results of the two scenarios are shown in Figure 8 and Figure 9.

The results show that the errors due to relative motion have been effectively eliminated. Along with the space delays and device delay correction methods mentioned above, we can infer that the system is widely applicable for the proposed operation modes and that this novel system can maintain a considerable accuracy of clock error extraction even for high-dynamic scenarios.

### 3.2. Clock Adjustment 

Once the clock error has been extracted, the next step is clock adjustment. In this simulation, the sub frame has an overlay of 0.2 s and the clock adjustment time period is 1 s, which implies that each control signal is predicted by 5 sets of previous data. The PLL parameters of the clock adjustment loop are described in Table 4. The bandwidth B_L_ of the control loop is 0.5 Hz.

**Figure 10:**
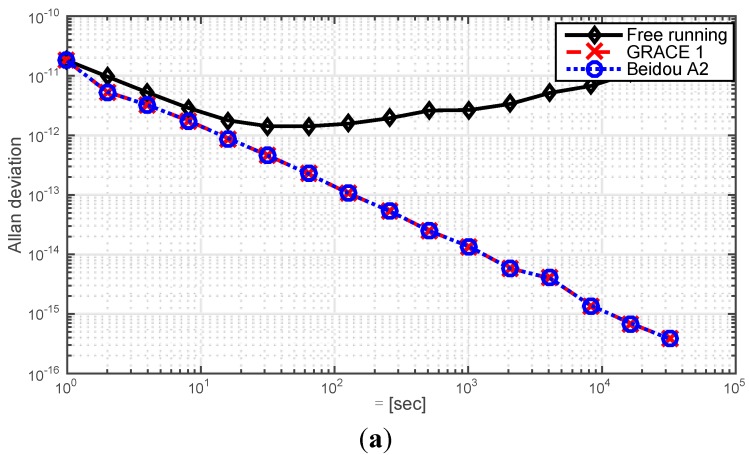

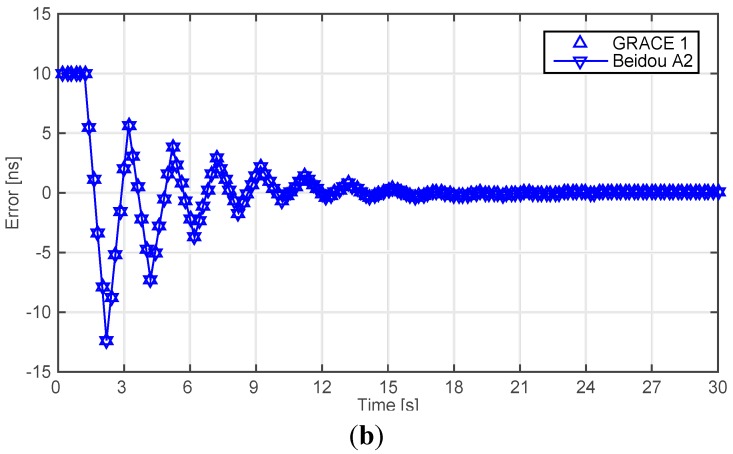
(**a**) Allan deviations of the simulated free running on-board VC-OCXO, and its Allan deviation in the provided scenarios when the synchronization loop stabilizes; (**b**) Time synchronization performance of GRACE 1 and Beidou A2.

For both two scenarios, the slave clock has the same on-board VC-OCXO. The free running characteristic of this particular clock is shown in Figure 10, it is obtained with *a*_0_ = 1 × 10^−8^ s, *a*_1_ = 1 × 10^−12^ s/s and $a_{2}=1\times 10^{-17}$ s/s^2^ and the free run clock 3 shown in Table 3 is employed.

Figure 10a shows the Allan deviations of the simulated slave clock in three different situations: free running and steered by the master clock in the GRACE and Beidou G1 & A2 scenarios. Compared with the free running clock, the Allan deviation reduces by orders of magnitude when the clock is steered. In addition, the Allan deviations of GRACE 1 and Beidou A2 are nearly the same, which proves that the dynamic error can be practically eliminated in both cases. To better express the performance of the proposed system, the simulated clock error of both scenarios are shown in Figure 10b, from which it can be seen that the synchronization loop stabilizes in less than 25 s, even in the event that the on-board clock is drifting. In order to evaluate the stability of the synchronization loop, statistics relating to the synchronization error data ${\text{ε}}_{n}$ from the first hour to the 24th h have been determined for an arbitrary number of initial conditions. These statistics include the bias $\text{Δ}\hat{x}=E\left[{\text{ε}}_{n}\right]$, the variance ${\text{σ}}_{\text{ε}}^{\text{2}}=E\left[{\left({\text{ε}}_{n}-\text{Δ}\hat{x}\right)}^{2}\right]$ root-mean-square deviation (RMSD), ${\text{σ}}_{\text{ε}}=\sqrt{{\text{σ}}_{\text{ε}}^{2}}$, and root-mean-square error (RMSE) ${\text{ε}}_{RMS}=\sqrt{E\left[{\text{ε}}_{n}^{2}\right]}=\sqrt{\text{Δ}{\hat{x}}^{2}+{\text{σ}}_{\text{ε}}^{\text{2}}}$. The results from these simulations are shown in Table 5.

**Table 5:** Synchronization errors of the GRACE mission and Beidou A2 & G1.

Synchronization Error (Picosecond)	Scenarios	
	GRACE	Beidou A2 & G1
Bias	0.280	0.280
RMSD	40.816	40.818
RMSE	40.817	40.819
Max error	20.86	20.87

We can therefore infer that the dynamic error and the clock error are effectively eliminated and that the system could meet the requirement of various scenarios with different relative velocities and orbit planes. 

### 3.3. Residual Errors

**Figure 11:**
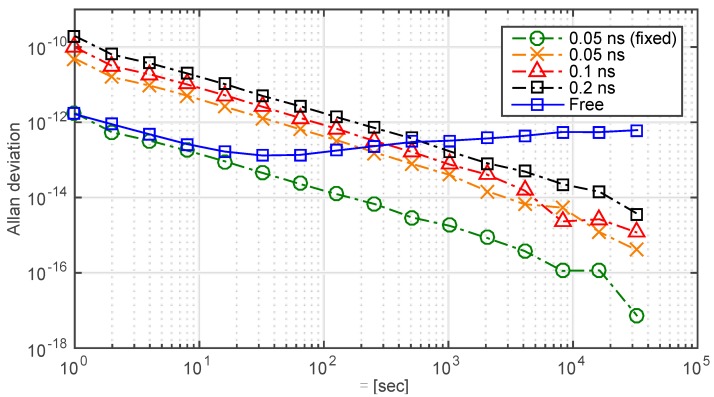
Comparison of the Allan deviations of a free run slave clock and a controlled clock for different residual errors. Data from the 1st hour to the 24th h are truncated to evaluate the synchronization performance. The standard deviations of the stable loops and the free run clock are 0.004 nanosecond, 012 nanosecond, 0.24 nanosecond, 0.47 nanosecond and 10 nanosecond, respectively.

The simulated synchronization performance previously described is expected to exceed the performance of an actual deployed system constructed with hardware & software owing to the degrading effects of the residuals. However, the residuals cannot be simulated unless that the specified hardware, frequencies and space scenario is confirmed. Therefore, in Figure 11, clock instability is analyzed for different residual errors, which are uniform randomly distributed in the range 0.05 nanoseconds, [−0.05, 0.05] nanoseconds, [−0.1, 0.1] nanoseconds, [−0.2, 0.2] nanoseconds and constant 0.05 nanoseconds, respectively. The bandwidth of the control loop is 0.5 Hz. It can be seen that the residual error has a great effect on the short-term stability of the slave clock when it is not a fixed value, which could be explained by that the PLL cannot get the absolute ideal control signal with the increasing instability of residual error.

### 3.4. Bandwidth of the Control Loop

**Figure 12:**
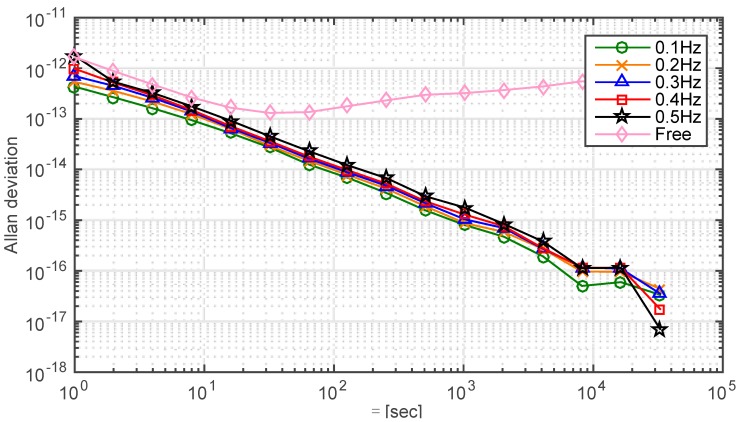
Comparison of the Allan deviations of a free run slave clock and a controlled clock for different control bandwidths. Data from the 1st hour to the 24th h are truncated to evaluate the synchronization performance. The standard deviations of the stable loops and the free run clock are 2.4 picosecond, 2.7 picosecond, 2.9 picosecond, 3.2 picosecond, 4.0 picosecond and 11 nanosecond, respectively.

The accuracy of the PLL depends on the bandwidth. Analysis of the behavior of bandwidth in Figure 12 shows that clock stability improves with the decreasing bandwidth. However, PLL bandwidth cannot be set arbitrarily small because the dynamic stress tolerance may be exceeded. Hence the performance of the clock must be taken into account when designing the bandwidth of PLL.

### 3.5. System Performance

**Figure 13:**
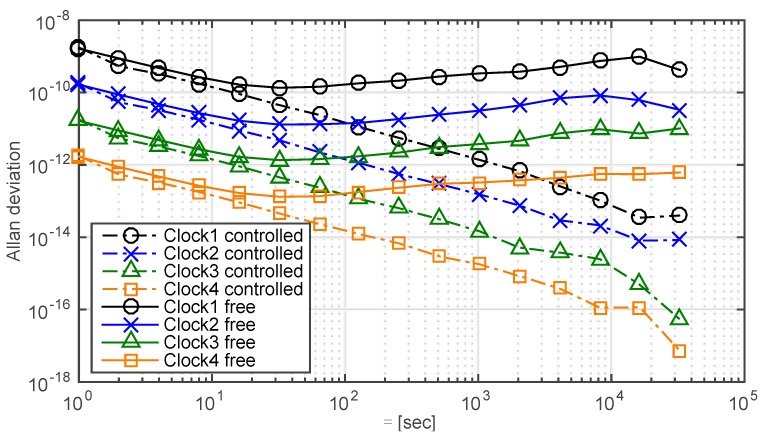
Comparison of the Allan deviations of four free run slave clocks and related controlled clocks. Data from the 1st h to the 24th h are truncated to evaluate the synchronization performance. The standard deviations of the stable loops and the free run clocks are 0.004 nanosecond, 0.04 nanosecond, 0.4 nanosecond, 4.1 nanosecond, 35.14 microsecond, 1.19 microsecond, 0.16 microsecond and 0.01 microsecond, respectively.

Now we consider the performance of the synchronization system, assuming a configuration with a single master clock and four slave clocks. Each of the slave clocks has a different level of performance, as indicated by the different free running Allan deviation plots shown in Figure 13, which also shows the controlled Allan deviations. The results imply that the slave clocks with different stability performances cannot be synchronized to a common master clock below a given threshold. In other words, the synchronization performance also relies on the free running stabilities of the slave clocks. Therefore, the same type on-board slave clocks should be applied for the proposed system in order to achieve better time synchronization performance.

It can be seen that some of the systems mentioned in Table 1 are real-life systems, while some are still in experimental stage. In addition, it goes without saying that the systems which employ two-way ranging would have a better accuracy of clock error measurement than the systems with one-way ranging. Furthermore, different clocks, clock adjustment intervals and space environments would affect the synchronization performances as well. Therefore, in order to fairly compare the proposed system with the other existing systems which also have the function of clock steering, the following assumptions are made.

**Figure 14:**
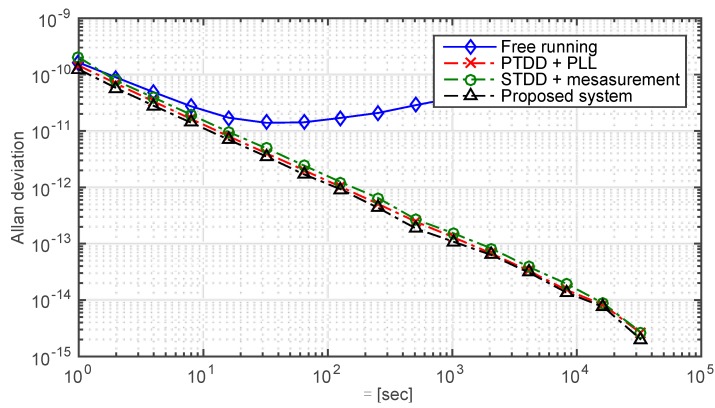
Comparison of the proposed scheme and two existed schemes. The Allan deviations of a free run slave clock and related controlled clock are presented. Data from the 1st h to the 24th hour are truncated to evaluate the synchronization performance. The standard deviations of the free run clock and the stable loops are 1.2 microsecond, 0.15 nanosecond, 0.18 nanosecond and 0.12 nanosecond, respectively.

First, all the systems adopt the same slave clock and the clock adjustment interval is 1 s. Second, all the systems are simulated under the same scenario. The PTDD scheme adopted by GPS and the scheme employed by NAMURU V3.2, which utilizes the measured clock error as the control signal are simulated, as shown in Figure 14. It can be seen that the proposed scheme has a better performance than the other schemes. We have reason to believe that the real-world performance would be better because of the implementation of the proposed phase error detection method.

## 4. Conclusions

A clock synchronization and syntonization system for satellites based on VC-OCXOs is described in this paper. Compared with the existed schemes, the proposed scheme behaves better under the similar simulated scenarios. A high-accuracy phase error extraction and correction method is proposed, including error analysis, correction methods and control signal prediction. The dedicated requirements for different scenarios are proposed. The simulation results imply that the proposed system could achieve a relative good synchronization and syntonization performance under the premise of reducing overall costs. The proposed dynamic compensation scheme proves that the system could reduce the dynamic error to sub-nanosecond level in various space scenarios, such as formation flying scenarios, GNSS constellations or hybrid constellations. The slave clocks are recommended to be the same type because the synchronization loop has a limited disciplining capability for a particular VC-OCXO. Moreover, the bandwidth of the control loop relates to the convergent rate and synchronization precision of the closed loop. In addition, the residual error is also a critical element that will affect the synchronization loop, which must be considered carefully. Although the key features and techniques for this novel system have been described in this paper, there still remain many interesting open issues, such as distributed synchronization loop design in case of holdover mode.
